# Interspecies Relationships in *Animal Crossing*’s Society: Utopia or Speciesism?

**DOI:** 10.3390/ani16131974

**Published:** 2026-06-26

**Authors:** Charlotte Duranton, Anaïs Perrin

**Affiliations:** 1 Laboratoire Textes et Culture, Université d’Artois, 62030 Artois, France; 2 Laboratoire Configurations Littéraires, Université de Strasbourg, 67000 Strasbourg, France; perrin.anais.ym@gmail.com

**Keywords:** *Animal Crossing*, video games, cultural studies, virtual society, interspecies relationships, animal ethics

## Abstract

*Animal Crossing* is a famous cozy video game in which players embody a human character living among anthropomorphic animals. Playing *Animal Crossing* has been evidenced to fulfill players’ Maslow’s needs, such as deficiency-motivated needs (safety, love and esteem—with the exception of physical needs) and growth-motivated needs (cognitive, esthetic, and self-actualization needs). We can suggest that animal characters, with their cute esthetic, are an important part of the game’s success. If such a virtual society is a safe place for humans (players and characters), what messages does it convey regarding our interactions with nature and other species? To answer this question, we investigated the relationships between players and nonhuman species in *Animal Crossing*: *New Horizons* and *Animal Crossing: Pocket Camp Complete*. Positive effects of such interspecies interactions have been observed at both intra- and extradiegetic levels: an improved knowledge of the animal kingdom and an improved sensitivity to its diversity. However, the game also has negative sides: its speciesist, colonialist and capitalist dynamics promote animals’ objectification. We then discuss how players can transform the game to consider all animal species with equal respect, and how other games with more ethical dynamics are a good model for further game designs.

## Simple Summary

*Animal Crossing* is a social simulation video game created by Nintendo. Players embody a human character living among various anthropomorphic animals, helping them get food and objects, or participating in group events. Its cozy gameplay aims at creating a feeling of belonging in players. Positive effects of such interspecies interactions have been observed at both intra- and extradiegetic levels: an improved knowledge of the animal kingdom, and a better sensitivity to nonhumans’ diversity. However, the game dynamics also have negative effects in virtual as well as real societies. Speciesist, colonialist and capitalist dynamics promote animals’ objectification, according to their economic value. Nevertheless, the game’s worldwide popularity is an efficient tool to raise awareness of nonhuman animals’ well-being.

## 1. Introduction

*Animal Crossing* is a social simulation video game series created by Nintendo, in which the player embodies a human character who lives among various anthropomorphic animals. Various versions of this “village simulator” [1] (p. 267) have been released since its creation: *Animal Crossing* (2001), *Animal Crossing: Wild World* (2005), *Animal Crossing: City Folk* (2008), *Animal Crossing: New Leaf* (2012), *Animal Crossing: Happy Home Designer* (2015), *Animal Crossing: Amiibo Festival* (2015), *Animal Crossing Pocket Camp* (2016), *Animal Crossing: New Horizons* (2020), and *Animal Crossing: Pocket Camp Complete* (2024). Furthermore, a significant update of *Animal Crossing New Horizons* (ACNH) was launched in January 2026, five years after the first release of the game. They all share a common principle in that the player creates a living space where many activities have to be done daily, such as picking fruits, fishing, searching for insects or fossils, and building furniture.

Characterized as kawaii (cute in Japanese culture) and sweet with a smooth world-building, *Animal Crossing* gameplay is thus the perfect example of a cozy game [2]. Additionally, its alignment with real-world time enables players to feel comfortable almost immediately [3]. Players have to repeatedly interact with villagers (ACNH) or campers (*Animal Crossing: Pocket Camp Complete* (PCC)) in order to get food, build furniture, or find lost objects. The game “provides a feeling of belonging to players” who define themselves as a “united, helpful, caring and generous community” [4]. Players can indeed interact together, through their characters, but also through social media and in real life. Numerous online forums gather players who love to share their experiences, creating spaces to socialize around the game. Players can visit each other’s islands, bridging the virtual world with real-life interaction. *Animal Crossing*’s aim is explicitly stated: its creator wants to fight the “pain from the disappearance of social connections, and the dangers of isolation and loneliness.” To do so, the game focuses on three main values: “family, friendship and community” [5,6] (p. 9), creating a strong sense of belonging. The dynamics of gameplay, more precisely interactions between different characters, have been studied extensively as a factor to explain such a phenomenon [6,7].

Playing *Animal Crossing* indeed fulfills Maslow’s needs, such as deficiency-motivated needs (safety, love and esteem—with the exception of physical needs) and growth-motivated needs (cognitive, esthetic, and self-actualization needs). Playing “in the virtual world has a role in satisfying needs in the real world” [8]. Many studies have identified the game as a safe space for players, for example, during pandemics such as COVID [4,9,10,11,12,13]. One reassuring effect is the predictability of each inhabitant’s behavior towards players. Rose indeed stated that “[t]o appeal to cozy ideals more powerfully, villagers are designed to embody different identities of cuteness and comfort predictably […]. Villagers are designed not only to be players’ friends, but predictable social assets, presenting as “an appealing means to experience comfort in a way that can be managed and controlled”” [14] (p. 7). Reported positive emotions also include friendship developed by players towards the inhabitants of *Animal Crossing*. Daniel Milne-Plückebaum [15] describes such a phenomenon as follows: “I’d like to tell you about some dear friends of mine […]. They’re friendly, charming and funny. Their quirky personalities make me laugh. I visit them almost daily. When I can’t catch up with them for a day, I miss them. What a friendship that is! I wonder what they’re up to right now? Hazel, the cute squirrel, who likes to drink her coffee black; Monique, the snobby cat, who wants to become a drummer; or Cyrano, the cranky anteater, who considers me his only friend!”. The researcher emphasizes the link between real feelings and fictional characters: “I fictionally act within the world of Animal Crossing, fictionally intend to help Monique and others, and fictionally feel sympathetic towards them when they fictionally tell me about what’s going on in their lives. I’ve actually developed certain feelings which can fictionally be regarded feelings of friendship” [15]. Predictability as well as friendly behaviors are thus suggested as the main factors explaining why players enjoy interacting with *Animal Crossing*’s citizens.

However, when considering *Animal Crossing*’s inhabitants, little attention has been paid to their specificities. All inhabitants, except for the players, are nonhuman animals. It is known that childhood has a particularly deep connection with animals [16], and we can suggest that animal characters, with their cute and kawaii esthetic, are an important part of the game’s success amongst younger players. Such a chibi (exaggerated cartoon style originated in Japan) design can also explain success amongst older players, as it is known that kawaii images elicit more care-like behaviors [17]. It has also been found that most players are females (75% [18]). This result is in line with the fact that women are more often associated with animal care both in real [19,20,21,22] and virtual life [23,24,25]. Additionally, the same study revealed that the average age of 15+ people playing *Animal Crossing* is 35 years old [18]. It is thus possible that people in this age group experience a genuine sense of nostalgia when interacting with animal characters. They might seek their reassuring, comforting company. But if such a virtual society is a safe place for humans (players and characters), what about others’ perspectives? What messages does the game convey regarding our interactions with nature and other species?

To answer these questions, we will investigate how human biases shape our relationships with nonhuman species in global cultural productions. We will focus on the imaginary interspecies shared society of *Animal Crossing*‘s virtual world. In order to study the game implications from cultural perspectives, we will focus our research on the two most contemporary versions: *New Horizons* (ACNH) and *Pocket Camp Complete* (PCC). However, occasional comparisons with previous versions of the game could be made to study the evolution of such a society and its present-day implications. From a literary perspective, we will focus our analyses on the game’s general settings and, most precisely, the interactions between players and non-player characters during gameplay. To do so, we will draw on previous scientific studies to build our theories, but will also consider empirical spontaneous players’ reactions on social media to support our statements. Firstly, we will study the positive effects of interactions with other animal species, at both intra- and extradiegetic levels. Then we will observe how the game’s dynamics actually have the opposite effect of erasing nonhuman beings from the studied society.

## 2. Positive Effects of Interspecies Interactions in *Animal Crossing*: Promoting Animal Identities at Both Intra- and Extradiegetic Levels

### 2.1. Virtual Society as a Tool for Educating People About Nature Protection in Real Life

Nature and its biodiversity are omnipresent in *Animal Crossing*’s universe. Players gather various organisms, such as fish, insects, fossils, or plants (fruits, flowers and trees), all designed after real-life species. They can also garden and redefine the whole island, “from creating paths and waterfalls to altering cliffs” [26]. The game has been evidenced to be an effective tool to learn about ecological concepts such as conservation and limitation of resources [27,28], as well as taxonomy [29]. For example, Blathers, a special character owl working as the Museum director and paleontologist, is “exceedingly interested in the ecosystem of this island!” in ACNH. When players dig fossils or catch animals (fish or insects), Blathers receives and identifies them (see, e.g., “After some consideration, I can safely declare this fossil to be a diplo tail!”) to ensure they can join the Museum’s official collection. Each time players bring an item, the bird asks: “Would you like to learn more about this specimen?”. Players can choose between “Yes, please tell me.” or “I’m a bit busy…”. If “yes” is selected, the owl provides a precise answer with scientific facts, such as “The Madagascan sunset moth is said to be the most beautiful moth in the world” or “The coelacanth is a deep-sea fish that has been around since the age of the dinosaurs. They were long thought extinct, so when living specimens were discovered, it was quite a shock!”. For two species of fishes (Napoleonfish and Asian arowana), Blathers’s explanations are directly related to real-life conservation issues—see e.g., for Napoleonfish: “Alas, this quirky creature is quite endangered due to a variety of factors. Needless to say, we must do our very best to take care of our lumpy friend and its environment.” Facts taught by the owl are indeed both accurate and useful in real life: “Through Blathers, players can therefore learn new information about biodiversity with positive implications for conservation awareness.” [27]. Interestingly, Blathers’s statements are, for example, used by PBS websites to educate people on butterflies. They report exact explanations given by the virtual owl and compare pictures of the game with real-life photos [30]. A study’s participant also testifies: “Since I’ve been playing in ACNH for a while […], planting and watering flowers, it [has] raised my interest in natural beauty, and I [’ve] started to like the flowers in real life. Now I’m more aware of plants and flowers in real life, and I even know their names. I’m very happy about this change.” [7]. Recent studies evidence such effects beyond empirical examples. Indeed, *Animal Crossing* players “are better than non-players at identifying real-life species that were present in the game” [28]. The authors concluded that representing various species in the game “impacts the ability to identify the species in real life”, “help enhance ecological learning” and “might be used as a tool for education in conservation biology” [29]. *Animal Crossing* also encourages other nature-protective behaviors. In ACNH, players can collect trash items, such as empty cans, lost boots or old tires, and then re-use them as recycled material for do-it-yourself recipes. The game thus promotes “pro-conservation behaviors and attitudes (e.g., recycling litter, or planting a diversity of flowers)” in players and is an efficient tool “to transform people’s relationship with the natural world.” [7].

**Figure 1:**
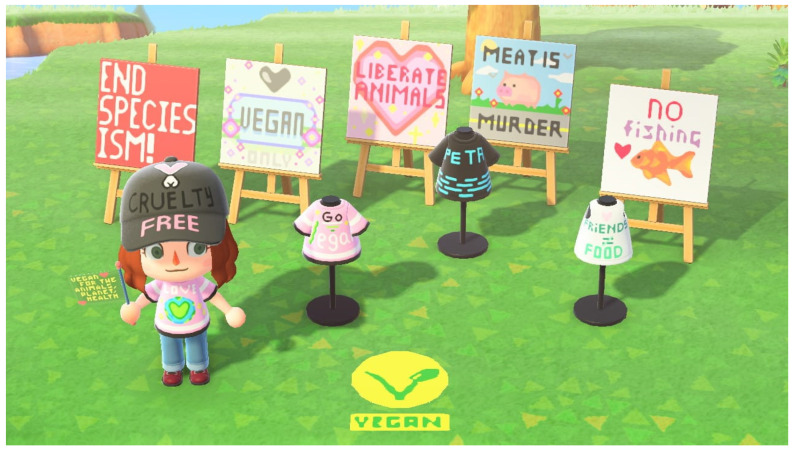
Examples of vegan custom designs made by players in ACNH (photo A. Perrin).

Based on such findings, as well as on the global fame of the game, charitable organization PETA (People for the Ethical Treatment of Animals) has created an online *Vegan Guide to Animal Crossing: New Horizons* [31] in which they give information on lesser-known species, much like Blathers does in the game. See, for example: “Clams are bivalves who may look less familiar to us than mammals, but they still deserve consideration and respect. They’re capable of a surprising range of behavior. Did you know, for instance, that they can escape from danger by burrowing through sand? Although it’s unclear whether they feel pain, in the real world, they play an important role in the ecosystem”, “in real life, hermit crabs can live for more than 30 years in their natural habitat on tropical seashores, but after being purchased in the pet trade, most don’t live for more than a few months to a year.” or finally “Tom Nook is a tanuki, or a raccoon dog, [they] are often killed for their fur. Others like him in the real world are beaten, anally electrocuted, gassed, or skinned alive. Cut him some slack.” [31]. PETA uses the ACNH universe not only to educate people on biodiversity, but also to transcend the game’s initial message. The organization questions some practices (such as fishing, catching invertebrates or even keeping pets in kennels) and encourages its readers to deconstruct such habits: “In the real world, animals suffer in captivity at places like SeaWorld and roadside zoos. They’re deprived of everything that’s natural and important to them. In *Animal Crossing*, you have the choice to let the animals on your island live free from harm, so please, leave them alone!” [31]. Finally, the organization also gives practical advice on how to use the game’s possibilities to understand veganism and act vegan if players want to: “Being vegan means not only not eating animals but also not wearing them. Avoiding products made with fur, leather, wool, down, or cashmere and opting instead for clothing made with vegan materials like cotton, microfiber, hemp, nylon, and polyester prevents animals from being slaughtered for fashion. In addition, wearing vegan is much better for the environment” or “How can you spread animal rights through Animal Crossing? Give your island an animal-friendly name like Veganville. Customize your passport with a phrase like #EndSpeciesism or “Adopt, don’t shop” or “Go vegan!” and “PETA hopes the game will encourage people to feel closer to the animals we share our planet with and inspire them to work to #EndSpeciesism—the misguided belief that humans are superior to all other animal species and that it’s OK for us to exploit some species in horrible ways for our own trivial purposes.” [31]. In their willingness to fight against speciesism, PETA uses some of the famous villagers to spread their message in an educational way: “Agnes, one of the starting villagers, is a sweet pig and a lovely neighbor. In real life, pigs are just as sweet, gentle, and kind. Mother pigs even sing to their young! But because of speciesism, humans slaughter pigs and eat their flesh, instead of recognizing them as sentient beings who deserve respect.” and “Another starting villager, Axel, is an elephant who loves to move and work out. Elephants in real life love to roam big open spaces with their families—but because of speciesism, humans capture, confine, and abuse them for entertainment. These magnificent animals are forced into a lifetime of misery for cruel rides and circus shows.” [31]. Additionally, in the same way that, once published, a book no longer belongs exclusively to its author but is shaped by its readers [32], the game itself has been reinterpreted by players. They create designs to express their opinions about other animals (see Figure 1) and invite people to consider them as free individuals with their own needs and personalities.

### 2.2. Virtual Interactions with Animals Enable Them to Express Their Own Personalities

We know that in both ACNH and PCC, players embody the only human character. In the village (ACNH) or the campsite (PCC), all other inhabitants belong to nonhuman species. Interestingly, players are the ones working for other animals, not the other way around. Players have various tasks to accomplish for their islands’ citizens: finding specific food items for them, helping them build their houses and furniture, helping them find lost items, etc. Humans are at the service of other species; there is no exploitation, forced obedience or any other use/mistreatment of any animal villager or camper, contrary to what happens in real societies (see, e.g., [33,34,35,36]). Moreover, to progress in the game, players must ensure the well-being of the inhabitants: “I chat with them, give them presents and write them letters, or take care of their little chores. I listen to their problems, stories and opinions.” [15]. Such a narrative structure is based on giving citizens their own personality, with preferences, behavioral and speaking habits, a proper esthetic… Villagers’ personalities are classified in eight different categories (see part II of the present paper for more detail about gender stereotypes associated with such personality traits), as follows:

- Lazy: male characters who are calm and hospitable, who love food and also have a laid-back lifestyle. There are 73 lazy villagers in ACNH.
- Jock: male characters who have a sportive or athletic nature. There are 78 jock villagers in ACNH.
- Cranky: male characters who are grumpy, easily annoyed and irritated. There are 66 cranky villagers in ACNH.
- Smug: male characters who are charming, kind, very polite and positive. They can sometimes flirt with the player. There are 38 smug villagers in ACNH.
- Normal: female characters who are sweet, kind and have neutral opinions. There are 77 normal villagers in ACNH.
- Peppy: female characters who are very friendly, bubbly and cheerful. There are 63 peppy villagers in ACNH.
- Snooty: female characters who are more mature and stylish, often wearing makeup. There are 67 snooty villagers in ACNH.
- Sisterly: female characters who are caring with the player but still very straightforward. There are 26 sisterly villagers in ACNH [37].

Besides those general traits, characters have their own storyline and individual history. They can also recall important social activities players did with them and use humor in their exchanges with players [7]. They use emotional language to create active interactions with players [38]. Players thus develop favorite characters, with whom they prefer to interact, called “dreamies” [39]. For example, a lot of social media videos are dedicated to such preferences, ranking inhabitants either according to the channel owner’s own views or based on surveys conducted among their followers (see, e.g., [40]). Overall, people prefer characters with a positive personality over pessimistic ones [41] but also appear to appreciate diversity in characters’ attitudes: “I liked him because it was kind of different” [41] or “Their quirky personalities make me laugh […]. Hazel, the cute squirrel, who likes to drink her coffee black; Monique, the snobby cat, who wants to become a drummer; or Cyrano, the cranky anteater, who considers me his only friend!” [15]. Finally, “[b]y populating your island with animals such as sheep, deer, and rabbits who have strong personalities, Nintendo is reinforcing the important fact that animals are individuals.” [31]. *Animal Crossing* redefines human–other species relationships. In the game, there is no hierarchy, no dominance, no abuse: other animals are villagers or campers. They are proper citizens, individuals with their own personalities. The franchise thus has a positive influence on the way interactions with other species can be represented.

### 2.3. Limitations

However, behind all these positive aspects, *Animal Crossing* gameplay conceals more problematic elements. For example, the above-mentioned inhabitants’ fixed personalities sometimes sound disturbing, with unnatural reactions. Such a phenomenon is empirically studied in *The Terrible Secret of Animal Crossing* [42], a short story based on the universe of the game. In 13 parts, the author tells the story of Billy, a young boy sent into the world of *Animal Crossing*, in a terrifying way: “There’s something seriously wrong here. Why aren’t there any other campers? […] I’m trapped here. And I’m alone.” (part 1)/“We get a new resident in town named Pate, and if you’re thinking he’s named after a food made from his own “species” you’re right.” (part 2, Pate is a duck)/“It occurred to me idly that I never saw any birds in this camp, never heard gulls crying on the beach.” (part 4)/“**Nook called it Animal Crossing**. Oh fuck. Oh my god. It all makes sense. This entire fucking camp is designed to keep me distracted!” “The longer you’re exposed to the gyroids, the more you cross over into some fuckep-up mutant animal creature! […] That’s why the island is full of animal-people…” (part 8). The reframing aims at revealing the game’s inconsistencies. Brown and Marklund suggested that *Animal Crossing*’s mechanisms—when interacting with the inhabitants—are similar to those evidenced by the literature studying horror games [43]. They imply three criteria: loss of agency (removing players’ sense of efficacy and control), the Freudian uncanny (a repeated experience between strange and familiar, and the existence of slightly distorted simulation of real-world behaviors in the villagers/campers), and the Heideggerian uncanny (ambiguities or incongruities in the perception of being/living, as the “inhabitants are ambiguously “alive””). Such mechanisms reinforce the players’ alienation from the game, and the novel “explores, through its mechanics, how terrifying society and individuals can be” [6] (p. 12).

Indeed, besides its apparent cuteness, *Animal Crossing* allows a deeper analysis of its society, and thus of real human societies. The game has been interpreted as an application of pastoralism ideology from Leo Marx, positing a link between pastoral—a literary model developing some nostalgic vision of the simplicity of life within nature—and pastoralist politics—a project organizing the world around this literary vision, with the same values [44]. The game may represent the ideal of a simpler life, closer to nature, free from industry and complexity. Pastoral ideology is characterized by the intrusion of technology into the naturalistic paradise [45,46]. Even if such a dichotomy is not explicitly present in the game, the opposition does exist between the virtual world, slow-paced and close to nature, and the real world, which is fast and dominated by technology. Such a feeling may explain the comforting side of the game but may also create “a sentimental manifestation of the pastoral ideal”, with an “almost fetishization of camping and natural reefs and national parks” [47]. Such a depiction conceals underlying game mechanisms rooted in colonialism and capitalist politics. *Animal Crossing* reflects, and at times even emphasizes, the contradictions in our relationship with the world, and particularly with animals. But what for? Does the game aim to debunk these contradictions, to expose them, or to raise awareness of such dysfunctions in real-world society?

## 3. Negative Effects of Interspecies Interactions in *Animal Crossing*: Intra and Extradiegetic Erasure of Nonhumans’ Individuality

### 3.1. Normalization of Speciesism and Unbalanced Human–Animal Relationships

The world of *Animal Crossing* appears to be a cozy place, fostering harmony with animals and nature. A closer look at the game’s mechanisms reveals the limits of such an idyllic picture. While there is a genuine sensitivity to nature, the pleasure of bending it to the players’ will, shaping it and exploiting it, is paradoxically central to the game. *Animal Crossing* establishes an asymmetrical approach to nature and animals, structured around humans’ will. Non-mammal animals (insects, fishes and marine creatures) are considered as objects, “critters”, cataloged in the game’s “Critterpedia”—an encyclopedia giving data on their seasonality and active hours to help players capture them. The game is therefore imbued with speciesism, defined by Horta as “the unjustified disadvantageous consideration or treatment of those who are not classified as belonging to a certain species.” [48]. The researcher later gave a more elaborate definition of speciesism, as “the unjustified comparatively worse consideration or treatment of those who are not classified as belonging to a certain species (or group of species) whose members are favored, or who are classified as belonging to a certain species (or group of species) whose members are disregarded.” [49], see also [50]. The general idea is that speciesism is a “discrimination against members of other species […] giving sentient beings of different species differing moral consideration for unjust reasons. This could be treating all nonhuman animals worse than humans, or it could be treating the animals of some species worse than animals of other species.” [51]. Speciesism can manifest in different ways, e.g., through anthropocentrism, anthropomorphism and non-anthropocentric speciesism. After defining each of them, we will observe how they are found in *Animal Crossing* and their consequences on human behavior.

Anthropocentrism “literally means human-centered” and is defined as “the ethical belief that humans alone possess intrinsic value. In contradistinction, all other beings hold value only in their ability to serve humans, or in their instrumental value.” [52] (p. 145). In *Animal Crossing*, anthropocentrism is easily observable and has been attested: players—the only human characters—hold all the power. The whole virtual society revolves around them and they have decision-making power over almost everything and everyone on the island. Players engage in “anthropocentric coziness, which grants them a complete control over their environment and allows them to indulge in the fantasy of a world in which there is ecological safety and stability.” [53] (p. 129). They also play “the dual roles of designer and beneficiary” [39] (pp. 74–75). By considering nature, including animals, as a way of maximizing players’ welfare, *Animal Crossing* promotes an anthropocentric dynamic. Nonhuman creatures, particularly critters but also inhabitants, are primarily useful to humans in both virtual and real societies. In *Animal Crossing*’s virtual world, critters are a way to earn money. Inhabitants contribute to the fulfillment of the players’ central objective, i.e., to create their ideal island, as well as to provide them with happiness and genuine support in real life.

Another speciesist side is found in *Animal Crossing*: anthropomorphism defined as erasing “animal’s animality, turning them into “stand-ins for humans”” [54] (p. 42). Such a process is a very common trope when it comes to representing animals, and villagers are their main representatives in *Animal Crossing*. They all have a very anthropomorphic lifestyle: they wear clothes and live in houses, they cook, build furniture, and practice sports. All of them eat fruits, insects, and fish as humans would, regardless of their species’ biological diet. For instance, in PCC, frog, rabbit or cow campers regularly ask for fish to eat. Even octopus campers, such as Marina or Octavian, sometimes request octopus to eat when they are hungry. Frog characters, such as Cousteau or Diva, can also ask for frogs as a meal. Additionally, anthropomorphized villagers in ACNH reflect essentializing stereotypes found in human societies. Gender stereotypes are easily observable, for example, with most of the female characters wearing makeup (e.g., Gloria, Tiffany, Muffy, Naomi). The gendered distribution of personality types is also stereotypical: Lazy, Jock, Cranky, and Smug for male characters, and Normal, Peppy, Snooty, and Big Sister for female characters. Finally, stereotypes associated with particular species are found: almost all gorillas have fitness as a hobby and Redd, the fox, is characterized by his trickery. Such anthropomorphism contributes to the erasure of animality, transforming nonhuman inhabitants into human-like avatars with a cute, plush-like esthetic. These representations encourage players to consider nonhuman animals from a human perspective. Moreover, it perpetuates stereotypes that may be detrimental: association with cunning has historically contributed to foxes’ bad reputation within our societies [55].

Non-anthropocentric speciesism, defined as the “preferential consideration of members of a certain nonhuman species over the members of other nonhuman species” [56], is the last form of speciesism found in *Animal Crossing*. It is considered “non-anthropocentric” since the human species is not directly involved, yet it remains anthropocentric because species are ranked according to their proximity to humans. *Animal Crossing* “creates a hierarchy of living creatures based on their anthropocentric value” and “[a]nimals with aesthetic and sentimental value, such as the anthropomorphic animals that inhabit the islands as villagers, are given special status” [53] (p. 128). Villagers are indeed mainly mammals: domestic species—such as dogs, cats, chicken, etc., and “charismatic megafauna” species—such as lions, tigers, eagles, elephants, wolves, bears, apes, etc. [57] (pp. 19–20). They are treated with more consideration compared to insects, fish, and marine creatures. The latter belong to species attracting less empathy as they are considered more distant from humans. For example, they are used as cooking ingredients in ACNH or food for the villagers in PCC. The game “construct[s] classes of certain animal species as open to killing” [58] (p. 179) [53] (p. 128). When not killed, they are captured and displayed in the galleries of Blathers’ Museum, next to fossils and paintings. The game turns them into objects to collect and gaze at. Players can visit the Museum as they would an aquarium or a zoo: “[t]hey proceed from cage to cage, not unlike visitors in art gallery who stop in front of one painting, and then move on to the next or the one after next.” [59] (p. 21). The Museum also normalizes small artificial enclosures. For example, tarantulas lie in a tiny square vivarium and Coelacanths, Oarfish and Spider crabs share a small tank. The game portrays the captivity of these animals as acceptable, even when their living conditions do not meet their biological needs [60]. Such practices affect players’ behaviors, as the permeability between our relationships with nonhuman beings in both the game and real life is clearly demonstrated by the “New Horizons Aquarium Tour” organized around the world [61]. It is not the only example of *Animal Crossing*’s use of the ambivalence in our relationships with nonhuman animals. Frogs, as a species, are a perfect example in ACNH: while there are frog inhabitants (such as Lily, Henry, and Frobert), there is also a frog critter. When players catch one, the catchphrase highlights this duality: “I caught a frog! Or it’s a new neighbor…and I have some apologizing to do.” The game thus normalizes some cognitive dissonance in human players which is also present in the real world [62,63,64], reflecting deeply rooted inequalities in the treatment of animals depending on their species. Thus, by structuring the entire game world around human players and by distinguishing anthropomorphized companions and animals that are consumed or displayed, *Animal Crossing* reproduces and reinforces speciesist biases in the relationship between human and nonhuman animals, in both virtual and real life.

### 3.2. How Colonialism and Capitalism Structure Our Interactions with Other Animals

As previously seen, one of *Animal Crossing*’s central goals is for players to build their ideal world on a desert island. Such a narrative brings on a colonial dynamic since human players are in a demiurgic position. They appropriate a “virgin” territory and shape it to their liking by exploiting natural resources. According to Alexi D. Smith, “the values that the game reinforces […] are rooted in colonialism” [65] (p. 129), as the ill-defined and featureless expanse around the island and its alleged desertion are “a common and convenient attitude for colonization” [65] (p. 130). Players take possession of the island and gradually shape it, choosing the location of each house and bringing in new inhabitants. They build bridges and ramps, plant flowers, and deforest areas as they please. Players’ power to shape the environment is at its highest in ACNH due to the new feature called “Island Designer”. Players can add or remove cliffs, divert waterways, add waterfalls, etc. *Animal Crossing* then encourages artificialization of natural environments by presenting it as positive and erasing all negative consequences for nonhuman life. Additionally, crafting and cooking mechanisms allow “exploitation colonialism” [65] (p. 131), reinforcing what Pinder calls the “extractivist colonial processes and narratives of Animal Crossing” [53] (p. 125). Players have to gather natural resources, including living beings, in order to produce food and items to collect or sell. Such a dynamic is similar to plundering. Presented as benign and rewarding, it normalizes extractivist colonialist practices in the real world. Moreover, *Animal Crossing*’s colonialist mindset is “rooted in attitudes of […] settler colonialism, in which settlers displace the indigenous peoples of a colonized area and establish a new and dominant permanent society” [65] (p. 130). Villagers are no exception to players’ modeling power [39]. Since the recent 3.0 update (January 2026), ACNH includes a hotel where new non-player characters can randomly visit players’ islands. Players’ reaction to such a new function reflects their attachment to controlling their inhabitants. Some players rejected tourists that they considered as “uglies” in opposition to the “dreamies” they want for their island [39] (p. 66) (e.g., see the videos labeled “Hotel is bad acnh” on TikTok [66]). Organizing a “colony” according to players’ preferences is obviously an important aspect of the game. Players’ statuses as “mayor” (NL), “resident representative” (ACNH) or “camp manager” (PCC) reflect their decision-making role on the island.

**Figure 2:**
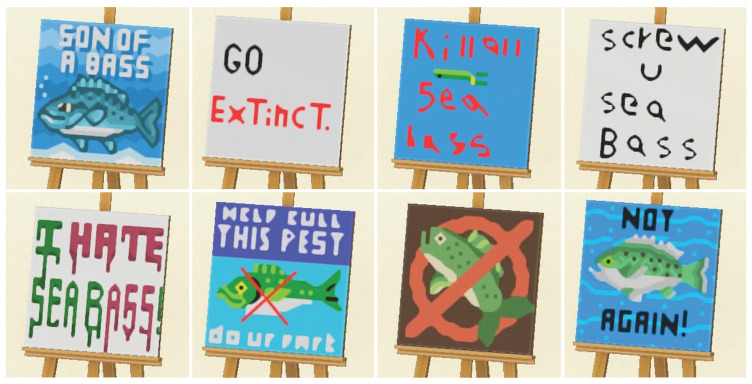
Examples of custom messages against sea bass in ACNH (photo A. Perrin).

*Animal Crossing* also relies on capitalist dynamics [6] (p. 96) as the game is built on “a procedural rhetoric of debt and consumption” [1] (p. 268). *Animal Crossing* has its own currency, “Bells”, and players start the game by taking out a loan from Tom Nook, the island/campsite’s “investor”. Players then have to collect and sell critters to make money and reimburse Tom Nook. The same process is repeated each time players want to build a new house/bridge on their islands. “By condensing all of the environment’s financial transactions into one flow between the player and Tom Nook, the game proceduralizes the redistribution of wealth in a manner even young children can understand. Tom Nook is a kind of condensation of the corporate bourgeoisie [1] (p. 270).” Additionally, almost everything has a price in Bells, including living beings. The capitalist dynamics of the game encourage players to capture and sell animals to make money, and to accumulate wealth to develop their island and home. Players’ interactions with nonhuman animals always have a financial dimension. For example, critters are similar to merchandise: capturing then selling them is one of the most effective ways to earn money in *Animal Crossing*. They can be stored in players’ inventories until sold (e.g., ACNH and PCC), given to villagers in exchange for money (e.g., PCC) or displayed like furniture (e.g., ACNH). To accumulate Bells faster, players have to repeatedly hunt and capture critters, in a farming dynamic, i.e., the “focused accumulation of resources from a single location” [67] (p. 178). *Animal Crossing* gamifies the wildlife business: players can repeatedly fish rare species in order to resell them and maximize profit. Players indeed target critters according to their financial value. Some species are favored, creating disregard for the less valuable ones. A notable example is the sea bass, which generates frustration for many players. The sea bass’s large size creates expectations of a rarer and more valuable catch. However, as a very common fish in the game, the sea bass has a low selling price. Players are regularly disappointed: “It is often considered as a constant annoyance to those who seek more valuable fish and mistake it for the only slightly larger blue marlin or coelacanth” [68]. Some players have even developed mods (i.e., user-created alterations to the game) to ensure the sea bass never appears in the game [69]. Other players have created virulent designs against the sea bass and share them in the game (see Figure 2).

The capitalist dynamics of *Animal Crossing* further reinforce the speciesism mentioned above. It normalizes living beings’ treatment as marketable goods within the game and, by extension, in the real world. One example is the “100% challenge”, which involves collecting all available items in the game, including critters. As *Animal Crossing* is an open-ended, exploratory sandbox game [70], players have been observed creating self-imposed challenges. When critters are not used to earn money, they become collector items, like artwork and furniture. Whether for profit or personal collection, each animal’s value is determined by its rarity. *Animal Crossing,* therefore, promotes a problematic model, as the harmful effects of rarity-based valuation have been observed in the real world. The “Anthropogenic Allee effect”, defined as “the human predisposition to place exaggerated value on rarity”, leads to “disproportionate exploitation of rare species, rendering them even rarer and thus more desirable, ultimately leading them into an extinction vortex.” [71]. Moreover, fishing, insect hunting and diving are unrestricted in the game: new critters always spawn for the player to capture them. Such a dynamic encourages intensive gameplay [65] (p. 131) but also obscures the risk of extinction in real life. As a result, critters are primarily framed as an inexhaustible resource, with their value determined by their price, which is itself tied to their rarity.

Capitalism also influences players’ relationships with anthropomorphic villagers (ACNH) or campers (PCC). Firstly, special characters, i.e., non-playable anthropomorphic animals who are not villagers, are mainly sellers. The most emblematic one is Tom Nook, a greedy tanuki, but all the characters function as specialized traders dealing in specific resources on the island or campsite. For example, Leif the sloth sells plants, Flick the chameleon buys bugs, and C.J. the beaver fishes. These characters embody capitalism’s prevalence in the game, as players constantly need to buy or sell living creatures to progress. Inhabitants are also affected by such capitalist logic. When players provide a service to a villager/camper, they receive objects or Bells as rewards. Players, therefore, never act without incentive but are instead driven by self-interested motivation. In PCC, quests to help campers appear every three hours and are the main way to obtain resources and money. At first, players must interact with campers to fulfill their requests. However, this quickly becomes “useless” as Pete the gull offers a delivery service that allows players to avoid “wasting” time on conversations while still earning Bells. A similar phenomenon can be observed in the option for players to delegate tasks to an assistant, a camper, who completes quests on their behalf while they are offline. The social and cooperative aspects of these quests diminish in favor of their financial aspect. Sincere relationships between humans and nonhumans are replaced by self-interested interactions: friendship with villagers becomes a means to accumulate items. Are “animals part of a collection, a perverse personal zoo, or do they have personalities the player can admire and even care about?” [1] (p. 275). It has been suggested that “sheer popularity of the most treasured villagers has even spurred grassroots marketplaces between players, in which both virtual and/or literal currency is traded for beloved villagers (players pay to visit others’ islands, which allow them to recruit dreamies).” [39] (p. 69). In ACNH, capitalism thus shapes players’ relationships with villagers, and instead of co-citizens, they become collectable items, in both virtual and real life. Nintendo has officially embraced such a market by selling trading cards that “can be collected for their own sake, or they can be used to insert the contents they depict on their faces into the game world” [1] (p. 274). Amiibo cards are well established in ACNH and even include crossovers with other licenses, such as Sanrio and Zelda. Players can therefore collect and buy specific villagers as they wish, much like furniture or clothing. Diegetic and extradiegetic forms of capitalism converge here, as in-game collection gives rise to real-world transactions involving actual money.

From critters to villagers, all relationships created with nonhuman animals in the game are distorted by colonial and capitalist logics. Beneath its naive and utopian appearance, *Animal Crossing* perpetuates anthropocentric and capitalist dynamics in which the reification and commodification of living beings are normalized and even rendered enjoyable. The game leaves little room for care, which is essential to develop more ethical human–animal relationships in real life [72]. Such representations resonate with and have consequences in the real world. The “immense popularity of ACNH means that the values put forward in the game are likely to influence society broadly” [65] (p. 130).

## 4. Discussion

*Animal Crossing* uses the inherent contradictions of our relationships with nonhuman animals. We have observed how capitalism, colonialism and speciesism are essential values in the game; however, it is important to remember the game’s positive impact. *Animal Crossing* offers freedom to players, with a sensitivity to nature. Such phenomena are a great opportunity to raise awareness and develop better human–animal relationships in our societies. See, for example, the case of charitable organization PETA, which created “PETA’s vegan guide to ACNH”, using the game’s popularity to raise awareness among citizens. PETA uses the catchphrase about hermit crabs—“I caught a hermit crab! I think it wanted to be left alone!”—to highlight the fact that players should not collect and sell them: “Don’t take hermit crabs from your island’s beaches and sell them to Blathers or Timmy Nook. They’re not objects—they’re individuals!” [31]. PETA also tries to bring to light the game’s non-anthropocentric speciesism: “It’s disappointing that a game in which villagers from all different species (from elephants and ducks to deer and pigs) coexist harmoniously with humans encourages abusive behavior toward fishes and insects. Instead of being recognized as the living, breathing, feeling individuals they are, they’re presented as forms of entertainment for the other villagers.” [31]. Interestingly, PETA organized an in-game protest where several human players protested in front of the Museum before storming into it, with slogans like “EMPTY THE TANKS” and “FISHES ARE FRIENDS”. The organization shared a video of its action on various social media platforms to spread awareness about animal welfare (see, e.g., [73]). Such an example allows us to suggest that video games, especially cozy games, are a good place to rethink our relationships with other species. We also suggest that animals found in the Critterpedia could be described in a more detailed way, including their role in our ecosystem, their conservation status, and what can be done to help/protect them.

To further understand such a phenomenon, we encourage researchers to investigate another sandbox cozy video game, *Stardew Valley* [74], aimed at the same audience as *Animal Crossing*. The game is an “open-ended country-life RPG” and offers players the chance to start a new life, following the seasons, in an old inherited farmhouse they have to restore [75]. Although *Stardew Valley* includes the possibility to raise and exploit nonhuman animals, it also offers players the choice to act differently. They can decide to focus their gameplay on plant production. Some players have even developed a vegan mod allowing game completion without the use of animal products: “I wanted to complete the game in a vegan way, no fishing no farm animals and no food with animal ingredients.” [76]. We thus encourage further research to explore such an add-on and its uses, to better understand how a more sensitive and egalitarian relationship with animals can shape new cultural practices in our societies.

However, some games are now designed from the outset with an ethical perspective that prioritizes the welfare of nonhuman animals. See for example PETA, which creates video games aimed at raising awareness about the condition of animals [77]. Some are parodic games, using famous names such as Nintendo’s *Mario*, *Pokemon*, *Super Meat Boy*, or *Cooking Mama*, respectively becoming *Mario Kills Tanooki* and *Super Chick Sisters*, *Pokemon Black and Blue—Gotta free’em all*, *Super Tofu Boy*, and *Cooking Mama: Mama Kills Animals*. The organization also created completely new games, such as *Kitten Squad* or *Whole Lotta Lies*, promoting animal liberation. Video games are thus an efficient tool to report issues concerning animal rights, as well as to change our relationships with nonhuman animals in real life. While not all games are as explicitly activist as the ones mentioned above, they still remain relevant subjects for study. *Terra Nil* is a cozy game “about transforming a barren, lifeless landscape into a thriving, vibrant ecosystem” and turning “dead soil into fertile grassland, clean polluted oceans, plant sprawling forest” to “create the ideal habitat for animals to call home” [78]. Although less famous than *Animal Crossing*, it has fewer anthropomorphic biases and would be a very good model to study. *Terra Nil* appears to be more ecocentric, as it genuinely places players in a role that serves Nature. We suggest that such gameplay would benefit animals by increasing players’ ethical concerns in real life. Additionally, part of the game’s profits are donated to the *Endangered Wildlife Trust* charitable organization. Positive effects on real animals are concrete and directly measurable by players, who are involved, even indirectly, in the preservation of wildlife. We thus encourage further research to explore the impacts of such a game on our relationships with nonhumans in our societies.

## 5. Conclusions

To conclude, we observed that *Animal Crossing*’s cozy utopia is largely influenced by power dynamics structuring human–animal relationships in real societies. *Animal Crossing* gives a significant place to nature and especially to animals, revealing their importance in the human mental constructions of utopia. However, such an apparent idyllic virtual society reproduces contradictions in our interactions with nonhumans. We can wonder if *Animal Crossing* voluntarily highlights such dissonance to help players reflect on their own ethical values. *Animal Crossing* “is morally ambiguous and open. It is up to the players to decide, to act according to their ethics, or to explore possible worlds that, nonetheless, are very much tied to the one outside the game.” [6] (p. 103). The present study thus evidenced how *Animal Crossing* reveals our fantasy of an ideal world shared with animals but remains structured and distorted by anthropocentric considerations. Nevertheless, it allows players to gain knowledge on fauna and hopefully to improve, however imperfectly, our relationship with other animals in the real world (bold is in the PETA publication): **“Take a break from your island life and help real world animals online today!**” [31].

## Data Availability

The original contributions presented in this study are included in the article. Further inquiries can be directed to the corresponding author.

## Abbreviations

The following abbreviations are used in this manuscript:

ACNH	*Animal Crossing: New Horizons*
AC:NL	*Animal Crossing: New Leaf*
PETA	People for the Ethical Treatment of Animals
PCC	*Animal Crossing: Pocket Camp Complete*
